# Effectiveness and cost-effectiveness of a 9 week multi-component cycling programme versus an existing single cycling training session: protocol for the Cycle Nation Communities randomised controlled trial

**DOI:** 10.1136/bmjopen-2025-112768

**Published:** 2026-03-03

**Authors:** Emma R. Lawlor, María Fernanda Gabler Trisotti, Emma McIntosh, Alex McConnachie, Jason M.R. Gill, Cindy M. Gray

**Affiliations:** 1 School of Cardiovascular and Metabolic Health, University of Glasgow, Glasgow, UK; 2 Health Economics and Health Technology Assessment, University of Glasgow, Glasgow, UK; 3 Robertson Centre for Biostatistics, University of Glasgow, Glasgow, UK; 4 School of Social and Political Sciences, University of Glasgow, Glasgow, UK

**Keywords:** Randomized Controlled Trial, Clinical Protocols, Behavior

## Abstract

**ABSTRACT:**

**Introduction:**

Cycling can be beneficial for health, well-being and the environment; however, cycling participation in the UK remains low. Effective and cost-effective strategies are needed to support people in the community to increase cycling. The Cycle Nation Communities randomised controlled trial (RCT) will evaluate whether a 9 week multi-component cycling programme (Cycle Nation) is more effective and cost-effective than an existing national cycle training session on cycling participation, transport use and health and well-being.

**Methods and analysis:**

This pragmatic, single-blinded, two-arm RCT will recruit ≥268 adults who cycle infrequently. Participants will be randomised to the 9 week multi-component individual/social-level group-based Cycle Nation programme or an existing national standard single group-based cycle training session. Both arms will be delivered by community-based cycling organisations in Glasgow. Participants will complete self-reported measurements at baseline, 12 weeks and 12 months. The primary outcome is the proportion of participants cycling at least weekly at 12 months. Secondary outcomes include proportion of participants cycling at least weekly at 12 weeks; change in weekly number of rides and minutes of cycling and use of private car, taxi, public transport and walking at 12 weeks and 12 months; change in motivation, perceptions of cycling safety, confidence to cycle, self-esteem, vitality, health-related quality of life and perceived general physical health at 12 weeks and 12 months. A within-trial economic evaluation from a National Health Service/personal social service and a broader societal perspective will be undertaken. Pending within-trial results, a long-term model may be developed. An embedded process evaluation will use participant and facilitator interviews, participant acceptability questionnaires, facilitator delivery proforma and session observations.

**Ethics and dissemination:**

Ethical approval has been obtained from the University of Glasgow Medical, Veterinary and Life Sciences Ethics Committee (11 April 25). Findings will be published in peer-reviewed journals and communicated to stakeholders and the public.

**Trial registration number:**

NCT07005674.

**STRENGTHS AND LIMITATIONS OF THIS STUDY**

- This is the largest randomised controlled trial (RCT) evaluating a multi-component behavioural cycling intervention which will provide robust evidence of effectiveness and cost-effectiveness.
- The RCT includes a cost-effectiveness analysis and embedded process evaluation.
- The 12 month follow-up is longer than most previous cycling intervention studies and enables assessment of sustained behavioural change.
- The Cycle Nation programme is delivered by community-based cycling organisations, supporting its real-world applicability and potential for scalability.
- All outcomes are self-reported.

## Introduction

 Cycling has substantial health benefits, including reduced risk of cardiovascular disease, type 2 diabetes, stroke, hypertension, several cancers, all-cause mortality and improvements in mental health.^1^,^5^ Investments in cycling have been shown to yield high cost–benefit ratios,^6 7^ foster social cohesion and community participation,^8^ and contribute to environmental sustainability by reducing air pollution, carbon emissions and traffic congestion through replacing motorised transport.^9^,^11^ Recognising these benefits, many governments, including the UK, have prioritised cycling as a key investment and policy action.

Cycling levels in the UK are low, with only 15% of adults cycling weekly.^11^ However, there is considerable latent demand, with 43% of adults reporting wanting to cycle more.^11^ This highlights a significant opportunity to increase cycling, provided that effective approaches to support participation are provided.

Previous evidence has identified that non-cyclists report multiple barriers to cycling, spanning individual, social and environmental domains of the socioecological model.^1 12^ Although environmental factors such as the provision of cycle lanes are important, evidence indicates that environmental change alone is insufficient to substantially increase uptake.^1 12 13^ Instead, participation is shaped by multiple levels, underscoring the importance of individual and social factors such as access to a bicycle, confidence and peer support.^1^

Community-based cycling organisations represent an opportunity to address these barriers, being well placed to deliver these interventions that are often more affordable and quicker to implement than large scale environmental changes.^12^ Many organisations already offer single or short-term cycling programmes which tend to address isolated components, such as a cycle skills training session, following the National Standard for Cycle Training. While addressing a key barrier to participation, this approach may not be sufficient to produce sustained long-term behaviour change. It is likely that more comprehensive, multi-component, longer term interventions addressing multiple barriers simultaneously would be more effective.^1 14 15^

Previous reviews have also highlighted important limitations in the evidence base. Many studies have small sample sizes and short follow-up periods (typically less than 6 months).^1 16^ Given the habitual nature of travel behaviour, with the need to embed into daily lives, short-term evaluations may underestimate the potential for sustained change.^16^ Furthermore, active travel intervention studies rarely include cost-effectiveness analyses, despite evidence that they can deliver positive returns on investment.^1 13^

The Cycle Nation intervention was initially co-developed and piloted in collaboration with British Cycling and HSBC UK as a workplace programme. This pilot study found that participants reported an average increase of 3.0 rides per week and 77% cycling at least weekly post-intervention, alongside improvements in perceived safety, vitality, confidence and motivation.^17^ Building on this success, the intervention was adapted for delivery in community settings in Manchester and Glasgow, where it demonstrated feasibility, acceptability and short-term effectiveness.

However, the Cycle Nation intervention has not yet been rigorously evaluated in a powered randomised controlled trial (RCT), nor has its long-term effectiveness or cost-effectiveness been assessed.

The Cycle Nation Communities RCT will evaluate whether a 9 week multi-component cycling programme (Cycle Nation) is more effective and cost-effective than an existing national standard single group-based cycle training session on cycling participation, transport use, and health and well-being. This will provide robust evidence on the additional benefits of a multi-component cycling programme beyond cycle training currently being offered, informing decisions about the wider implementation of the intervention and its potential to deliver health, environmental and economic benefits.

### Objectives

#### Primary objective

The primary objective is to evaluate the effect of the Cycle Nation intervention versus an existing national standard single group-based cycle training session on the proportion of participants cycling at least weekly at 12 months.

#### Secondary objectives

To evaluate the effect of the Cycle Nation intervention versus an existing national standard single group-based cycle training session on:

- Proportion of participants reporting cycling at least weekly at 12 weeks.
- Number of weekly cycling journeys (overall, leisure, commuting, errands) at 12 weeks and 12 months.
- Total duration of weekly cycling (overall, leisure, commuting, errands) at 12 weeks and 12 months.
- Number and total duration of weekly private car/taxi/public transport journeys at 12 weeks and 12 months.
- Number and total duration of weekly walking journeys at 12 weeks and 12 months.
- Self-reported motivation to cycle, perceptions of cycling safety, confidence to cycle, physical activity, self-esteem, perceptions of the environment, vitality, well-being and health-related quality of life at 12 weeks and 12 months.

To evaluate the within-trial cost-effectiveness of the Cycle Nation programme at 12 months (and longer pending within-trial findings).

To assess the fidelity and quality of Cycle Nation’s delivery, and how its core mechanism’s function.

To determine how contextual factors influence engagement, outcomes and variation across participating organisations.

## Methods

This protocol was developed and reported in accordance with the Standard Protocol Items: Recommendations for Interventional Trials (SPIRIT) 2025 statement.^18^

### Trial design

This will be a pragmatic, randomised, two-arm, parallel group, superiority RCT. Following baseline measurements, participants will be individually randomised using a 1:1 allocation to either the Cycle Nation intervention or to an existing national standard single group-based cycle training session. Participants will be allocated a specific delivery organisation according to preferred location, time and availability. The study is part of the GALLANT (Glasgow as a Living Lab Accelerating Novel Transformation) project, a large-scale city-university partnership using Glasgow as a real-world laboratory to test sustainable solutions to address climate change.^19^

### Eligibility criteria

A minimum of 268 participants will be recruited. Inclusion criteria will be as follows: aged ≥18 years; able to ride a bicycle and currently cycling less than once per month (see table 1).

**Table 1:** Participant inclusion and exclusion criteria

Inclusion criteria	Exclusion criteria
Can ride a bicycle Self-identified infrequent cyclists (currently cycle less than once per month) Aged 18 years or over Willing to provide informed consent Willing to complete questionnaires Willing to be randomised to intervention or comparison arm Have a good understanding of the English language No contraindication to moderate intensity physical activity as assessed by the adapted Physical Activity Readiness Questionnaire-Plus (PAR-Q+) (or provides an email with medical professional approval) Able to attend in-person sessions (at least 6 out of 9 sessions) Intend to still be in the UK by November 2026	Cannot cycle (ie, cannot balance and/or have never learnt to cycle) Frequent or regular cyclists (ie, currently cycle at least once a month, confident riding on roads) Less than 18 years old Not willing to provide informed consent Not willing to complete questionnaires Not willing to be randomised to the intervention or control arm Cannot speak basic English Contraindication to moderate intensity physical activity as assessed by the adapted Physical Activity Readiness Questionnaire-Plus (PAR-Q+) Not able to attend in-person sessions (less than 6 out of 9 sessions) Does not intend to still be in the UK by November 2026

### Trial setting

The intervention and comparator programmes will be delivered by trained facilitators from Glasgow-based commercial or third sector cycling organisations. Facilitators may work across multiple organisations; however, each facilitator will only deliver either of the programmes to avoid contamination.

### Intervention: Cycle Nation

#### Content and structure

Cycle Nation is a multi-component individual- and social-level cycling intervention consisting of nine, weekly interactive sessions, each lasting approximately 90 minutes, delivered to groups of up to 10 participants. Sessions will be led by two trained cycle facilitators covering off-road and then on-road cycle skills training as well as cycle maintenance training (eg, fixing a dropped chain), information-sharing activities and behaviour change techniques (eg, weekly goal setting). Two optional 90 minute ‘catch-up’ sessions will be offered to any group in which more than two participants have missed three or more sessions by the end of session 7. These sessions are designed to help participants feel safe and confident on the roads. Detailed content is available in table 2.

**Table 2:** Content of Cycle Nation programme

Session (week)	Discussion and behaviour change techniques	Practical	Practice ‘in own time’
0: Collect bicycle and equipment	N/A	N/A	N/A
1: On your bike	Overview of Cycle Nation Training Why Cycle?	Helmet, lights and lock ABC check Adjusting seat height Starting and stopping effectively	Lock your bike ABC check Ride your bike
2: Going through the gears	Review of ‘in your own time’ tasks What to wear Introduction to personal cycling goals	ABC check Going through the gears	ABC check Ride your bike Try out changing your gears
3: Basic cycling skills	Review of ‘in your own time’ tasks A brief introduction to barriers Buying a bike Personal cycling goals	Fixing a dropped chain Bike handling—cornering	ABC check Ride your bike Fix a dropped chain
4: Consolidating cycling skills	Review of ‘in your own time’ tasks Personal cycling goals	Pumping tyres Avoiding obstacles Front wheel lift Group (off-road) riding	ABC check Ride your bike Pump tyres Front wheel lift
5: Using the road	Review of ‘in your own time’ tasks Involving others Your rights on the road Personal cycling goals	Signalling Emergency stop Introduction to on-road riding	ABC check Ride your bike Practice signalling and emergency stops
6: On the road again	Review of ‘in your own time’ tasks Overcoming barriers Personal cycling goals	Lubricating the chain Traffic lights and roundabouts Filtering through queued traffic	ABC check Ride your bike Practise traffic lights
7: Puncture clinic	Review of ‘in your own time’ tasks Personal cycling goals	Replacing an inner tube	ABC check Ride your bike Change your inner tube
8: Planning a journey	Review of ‘in your own time’ tasks Overcoming setbacks Route planning Long term bike access Personal cycling goals	Riding a planned journey	ABC check Ride your bike Plan a group ride
9: Moving forwards	Review of ‘in your own time’ tasks Action planning and moving forwards Graduation	Group ride	Keep riding
10 & 11: ‘Catch up’ (optional)	Welcome back and review Your rights on the road Personal cycling goals	Signalling Road positioning Traffic lights Filtering through traffic Roundabouts	Keep riding

ABC, air, brakes, chain; N/A, not applicable.

Rides between sessions: cycle facilitators will organise two additional led rides (approximately 90 minutes) between the sessions from weeks 5 to 9 once participants have progressed to on-road cycling. Participants will be encouraged to cycle between sessions to embed cycling into daily life, including meeting up with other group members to practise skills and build confidence.

WhatsApp: participants will be invited to join a WhatsApp group with the facilitators and group members. This will help to build peer support, with the facilitators sharing motivational messages to encourage cycling between, and attendance at, sessions.

Bicycle provision and equipment: participants will be loaned a bicycle, helmet, lock and lights for the duration of the intervention to reduce financial barriers and support engagement, especially for those uncertain about taking up cycling. Participants will receive a handbook covering course content.

#### Delivery

Cycle Nation sessions will be delivered by facilitators from eight Glasgow-based cycling community-based organisations. Facilitators will be cyclists with previous experience in delivering group rides who have attended a 2 day interactive Cycle Nation training course led by a British Cycling trainer and a University of Glasgow researcher.^17^ Training includes practical instruction in session delivery, including supporting interactive group discussions, behaviour change techniques and leading group rides. Facilitators will be provided with a manual containing detailed session plans.

At the end of the programme, participants will be signposted to further support such as cycle maintenance classes or local cycling groups to promote long-term cycling engagement.

### Comparator: single group-based cycle training session

This will be an existing single training session (‘Introduction to On-Road Cycling’) linked to the National Standard for Cycle Training and delivered by one community-based organisation.^20^

This existing ~2 hour session targets adults who can already cycle and want to build the skills and confidence to ride on road. The session will focus on essential cycling skills, including hazard awareness and the key skills of observation, road positioning, effective communication and identifying road priorities, tailored to participants’ abilities. Up to six participants will be accommodated at each session and will be delivered by one certified instructor.

### Recruitment

The study will be publicised in community venues across Glasgow, for example, posters in local libraries, community centres and health centres, and cycling organisations. Furthermore, it will be advertised through GALLANT project email lists, partner organisations and social media.

### Enrolment and consent

#### Participants

**Figure 1:**
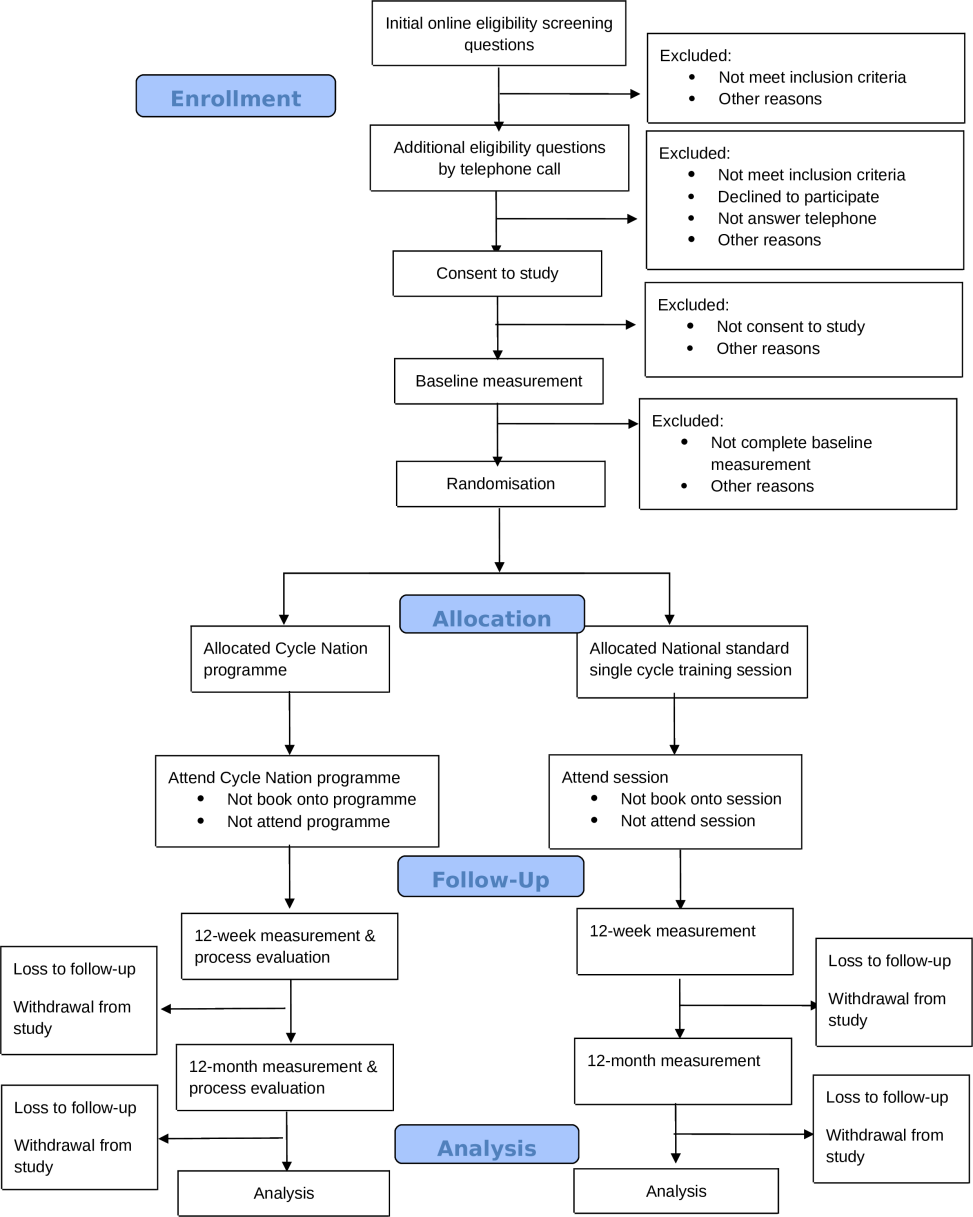
CONSORT diagram of participants. CONSORT, CONsolidated Standards Of Reporting Trials.

Potential participants will register their interest online via a link or QR code with brief screening questions: 1. ‘Are you 18 or over?’; 2. ‘Can you ride a bike?’; 3. ‘Have you cycled 10 times or less in the past 6 months?’. Those who meet these initial criteria will be prompted to provide contact details. Ineligible individuals will be notified automatically. Potentially eligible participants will be sent the study documents. After 24 hours, a researcher or fieldworker will contact them by phone to explain the study in detail, answer questions and conduct additional eligibility screening questions (see table 1). Reasons for ineligibility will be recorded. Eligible participants will be verbally consented and enrolled in the study (see figure 1 and online supplemental material 1 for copy of consent form).

#### Cycle facilitators

Facilitators delivering the Cycle Nation intervention will be consented in person before delivering the first session using physical consent forms (see online supplemental material 2 for copy of consent form).

### Data collection methods

All participants who consented to the trial will be emailed a link to complete a 30 minute self-report questionnaire online hosted on the Qualtrics platform (Qualtrics, Provo, UT) at baseline, 12 weeks (post-programme) and 12 months (see table 3). A £100 voucher will be provided to those completing all time points, and home visits may be offered at follow-up time points to reduce loss to follow-up.

**Table 3:** Participant timeline: schedule of enrolment, interventions and assessments

	Trial period				
	Enrolment	Baseline and randomisation	Post-randomisation		
Timepoint			Start of intervention (from week 1)	~12 weeks from baseline	12 months from baseline
Enrolment					
Eligibility screen	X				
Informed consent	X				
Randomisation		X			
Intervention/comparator					
Cycle Nation			x 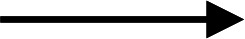		
National standard single cycle training session			x 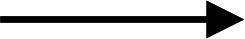		
Assessments (questionnaires)					
Sociodemographic characteristics		X			
Local environment		X			
Previous cycling injuries		X		x	x
Cycling levels		X		X	X
Motorised transport use		X		X	X
Walking levels		X		X	X
Perceptions of cycling safety		X		X	X
Perceptions of environment		X		X	X
Confidence to cycle		X		X	X
Motivation for cycling (BREQ-2)		X		X	X
Self-esteem		X		X	X
Vitality		X		X	X
Health-related quality of life (EQ-5D-5L)		X		X	X
Capability well-being (ICECAP-A)		X		X	X
Delivery and acceptability questionnaire (Cycle Nation participants)				X	x
Interviews					
Participants				X	X
Non-completers				X	
Cycle facilitators				X	

BREQ-2, Behavioural Regulation in Exercise Questionnaire; EQ-5D-5L, EuroQol 5 Dimension 5 Level; ICECAP-A, ICEpop CAPability measure for Adults.

### Outcomes

#### Primary outcome

The proportion of participants reporting cycling for any purpose at least weekly (ie, at least four times in the previous 4 weeks) at 12 months. This will be assessed using a question on number of cycling journeys (‘Over the past four weeks, how many times have you cycled for leisure or exercise/commuting/errands or go from place to place?’)

#### Secondary outcomes

Cycling: the proportion of participants reporting cycling for any purpose at least weekly (ie, at least four times in the previous 4 weeks) at 12 weeks, and changes from baseline to 12 weeks and 12 months in the number of weekly journeys and total duration of weekly cycling journeys (minutes) over the past 4 weeks.

Motorised transport and walking: changes from baseline to 12 weeks and 12 months in the number of weekly journeys and total duration of weekly private car/taxi/public transport or walking journeys (minutes) over the past 4 weeks.

Perceptions of the environment for safe cycling, well-being, physical activity, self-esteem, vitality and motivation: changes from baseline to 12 weeks and 12 months in:

- Perceptions of cycling safely measured using six statements related to road safety policies. Participants will be asked how safe they feel when cycling, selecting all that apply. They are asked to add additional road safety policies.
- Perceptions of the local environment measured using 13 statements relating to how participants feel on local roads,^21^ each requiring a response on a 5-point scale (ranging from strongly agree to strongly disagree).
- Confidence to cycle measured using seven statements concerned with cycling confidence,^22^ each requiring a response on a 5-point scale (ranging from ‘strongly disagree’ or ‘strongly agree’).
- Health-related quality of life measured using the EuroQol 5 Dimension 5 Level (EQ-5D-5L).^23^ Participants will rate their mobility, self-care, usual activities, pain/discomfort and anxiety/depression on a 5-point scale. Participants will rate their health today on a visual analogue scale from 0 to 100.
- Capability well-being measured using the ICECAP-A (ICEpop CAPability measure for Adults).^24^ This includes five statements related to Attachment, Stability, Achievement, Enjoyment and Autonomy, each requiring a response from four options.
- Self-reported physical activity measured using the Short Form International Physical Activity Questionnaire (SF-IPAQ),^25^ assessing moderate and vigorous intensity physical activity. The questionnaire consists of seven questions considering time spent being physically active in the past 7 days.
- Self-esteem measured by the 10-item version of the Rosenberg self-esteem questionnaire.^26^ Participants will rate each statement on a 4-point Likert scale (ranging from ‘strongly agree’ to ‘strongly disagree’).
- Vitality measured using the Subjective Vitality Scale,^27^ with six statements on a 7-point scale (ranging from ‘not at all true for me’ to ‘very true for me’).
- Motivation for cycling measured using the adapted Behavioural Regulation in Exercise Questionnaire (BREQ-2/3),^28^ measuring participants’ intrinsic motivation, identified regulation, introjected regulation, external regulation and amotivation for exercise. The questionnaire comprises 15 statements, each requiring a response on a 5-point scale (ranging from ‘not true for me’ to ‘very true for me’).

#### Local environment

Questions will be adapted from the ‘ALPHA measure of environmental perceptions: active travel and physical activity’^29^ to assess perceptions of neighbourhood, home environment and workplace/study environment at baseline. Participants at 12 week and 12 month follow-up will be asked if they have moved home since baseline, with any that have requested to complete these questions again.

#### Adverse events

Cycle facilitators will take note of any accidents/injuries that happen during and between sessions and report them to the research team. Accidents or injuries will also be assessed by asking the question at 12 week and 12 month follow-up, ‘Have you had any accidents or injuries related to cycling since starting the programme?’. Participants select yes/no and are asked to provide detail if yes.

#### Sociodemographic measures

Participant characteristics (eg, date of birth, gender, ethnicity, marital status, education, employment status and income) will be collected at baseline. Residential postcode will be recorded at baseline, with participants asked at 12 week and 12 month follow-up whether they have moved residence, with those who have asked to provide their new postcode.

### Economic evaluation

The economic evaluation will explore the cost-effectiveness, cost-utility and cost-consequences of the Cycle Nation intervention compared with the existing national standard single group-based cycle training session over 12 months and following a prespecified Health Economics Analysis Plan. The resources required to deliver the Cycle Nation intervention and the standard session will be identified, measured and valued, and include:

- Facilitator training costs.
- Participants’ cycle training.
- Training programme delivery costs.
- Any facilities costs incurred.
- Materials provided for intervention costs.
- Capital items (eg, cost of bikes/bike loan; helmets and locks).
- Reusable.

Resource use data will be combined with unit costs from national databases and routine sources relative to a reference year. Within-trial healthcare, social care and personal resource use data will be measured through the outcome questionnaire to identify frequency and costs related to:

- General practitioner visits.
- Nurse visits.
- Hospitalisations (including Attendances and Emergency and out-patient).
- Over the counter medications and lifestyle products (eg, slimming aids).
- Costs of gym membership and/or classes.
- Costs of previously purchased fitness/health apps.
- Average time spent exercising and walking daily.
- Cycling frequency and distance.
- Employment.
- Total annual household income.

For the economic outcomes, Quality Adjusted Life Years (QALYs) will be derived using the EQ-5D-5L (21) instrument combined with within-trial duration (months) and the Years in Full Capability (YFC) will be derived using the ICECAP_A instrument (25) combined with within-trial duration (months).

A National Health Service and personal social service perspective will be used for the base case economic evaluation. Sensitivity analyses using a broader societal perspective will also be conducted.

### Process evaluation

A mixed-methods process evaluation, embedded within the RCT and guided by the MRC Process Evaluation Framework,^30^ will examine the reach, delivery, mechanisms of impact and contextual influences of the Cycle Nation intervention. The evaluation will explore potential unintended consequences and variation in delivery across participating community cycling organisations.

Participant interviews: In-depth semistructured interviews will be conducted with approximately 25 participants, with one to two participants from each Cycle Nation group (45–60 minutes, by telephone or using online software). The same participants will be interviewed at both 12 week and 12 month follow-up to explore mechanisms of change and factors influencing sustained cycling. In addition, all non-completers (defined as attending fewer than six sessions or reporting discontinuation) will be invited to a shorter interview (approximately 30 minutes) to explore reasons for non-completion and barriers to engagement.

Questionnaires: A brief post-intervention participant questionnaire will assess the acceptability of programme content, perceptions of facilitators and delivery venue, and cycling equipment. At the 12 month follow-up, additional questions will explore sustained use of programme strategies and ongoing contact with peers and facilitators. These questions will be embedded within the outcome questionnaires for intervention participants.

Facilitator perspectives: Cycle Nation facilitators will participate in interviews or focus groups (45–60 minutes, in person, by telephone or online) after they have finished delivering all their allocated Cycle Nation groups to capture experiences of programme delivery, supporting participant engagement and perceived barriers and facilitators to implementation. Facilitators will complete a structured proforma immediately after each session to record fidelity of delivery, time spent on preparation and a description of how and why any key delivery points were not delivered as intended.

Observations: Two sessions per group will be observed by trained fieldworkers, including session four for most groups and one additional session sampled across groups. Observations will include items on fidelity of session delivery and field notes to provide a thick description of fidelity, delivery style and participant engagement.

#### Uptake, adherence and retention

The research team will record the number of individuals who express interest, are eligible, consent and are randomised to an intervention. Reasons for withdrawal will be recorded, if provided.

The cycle facilitators will submit weekly attendance registers via Microsoft Teams. Reasons for missed sessions will be collected, and attendance at six sessions, including ‘catch-ups’ when required, will define completion. Comparator group attendance at the single session will also be recorded.

### Sample size calculation

The primary outcome is the proportion of participants reporting cycling for any purpose at least weekly at 12 months. The study has been powered to detect a difference of 2 in 10 people cycling at least weekly between groups (ie, 10% vs 30%, 20% vs 40%, 30% vs 50%, 40% vs 60%, etc). A sample size of 214 participants (107 per group) provides 80% power to detect a 20% absolute difference in cycling prevalence (40% vs 60%) and a 0.38 SD difference in continuous outcomes at a 5% significance level. To account for an anticipated 20% loss to follow-up, the sample size will be inflated by 25%, yielding a maximum of 268 participants (134 per group). Participants enrolling together will be treated as a single randomisation unit; few such clusters are expected, and their impact on statistical power is minimal.

### Randomisation

#### Sequence generation, allocation concealment mechanism and implementation

Randomisation to the intervention and comparator group will be predominantly simple individual (1:1). However, during screening, participants will be asked if they know anyone else (eg, friends, family) who is planning to join the study. If the other person is eligible and enrolled within 7 days, they will be randomised together to reduce contamination. This small-scale clustering is factored into sample size and analysis. The randomisation sequence will be computer generated and implemented via a telephone interactive voice response system. Participant enrolment and randomisation to intervention or comparator will be undertaken by members of the research team and fieldworkers.

#### Blinding

Participants cannot be blinded to their allocation due to the nature of the intervention. Researchers undertaking the statistical analyses will be blinded to group allocation. There will be no circumstances in which the participants’ allocation will be revealed to the analysis team prior to analysis. Other study team members will not be blinded as they will be involved in allocating participants to intervention deliveries and conducting session observations and interviews.

### Data management

A member of the research team will review the data after a participant has completed a questionnaire and will contact participants if there are any unusual entries (eg, unexpectedly high/low values) to ensure data quality. Data will be exported from Qualtrics and saved securely on a University of Glasgow study server. A study-specific data management plan has been developed and submitted to the University of Glasgow Data Protection team.

### Statistical analysis

#### Outcome evaluation

A full statistical analysis plan (SAP) will be finalised prior to any unblinded analyses. The Roberston Centre for Biostatistics (Glasgow Clinical Trials Unit), University of Glasgow, will provide statistical services in support of delivering the trial.

The primary outcome will be compared between groups using a mixed effects logistic regression model, allowing for clustering and adjusting for important baseline characteristics, and reported as an adjusted OR, with 95% CI and p value. Absolute differences will also be reported. Analyses will follow intention-to-treat principles. Appropriate regression-based methods will be applied to secondary outcome measures. Missing data will be managed using multiple imputation with chained equations, applied separately within groups. Complier average causal effects methods will be applied to estimate the effects of the intervention for those who engage, as well as the effects of increased cycling on other outcomes. Sensitivity analyses will determine the association between attendance at intervention sessions and effectiveness. Subgroup analyses will be pre-defined in the SAP.

#### Economic evaluation

Within-trial results for the cost-utility and cost-effectiveness analyses will be presented as mean cost, mean effect and incremental cost-effectiveness ratios (ICER), that is, the incremental cost-per-unit of increase in the proportion of participants cycling at least weekly. For all economic analyses, the mean cost and mean outcome (increase in cycling, QALYs and YFC) associated with the intervention and comparator groups will be estimated using a generalised linear model, which will tackle the non-normality of data, adjusting for relevant covariates (eg, demographics). Non-parametric bootstrapping techniques will explore uncertainty around estimated costs, outcomes and the ICER. Costs, combined with QALYs, will be presented on the incremental cost-effectiveness plane.^31^ Joint uncertainty in costs and outcomes will be represented on a cost-effectiveness acceptability curve and the prevailing UK willingness-to-pay thresholds for cost-effectiveness will be employed.^32^ A multiple imputation procedure using chained equations will be used to impute missing outcomes and resource use separately for each arm of the trial, and predictive mean matching will allow dealing with the non-normality of cost and outcome data.^33^ The economic evaluation will be reported and presented in line with current economic evaluation methods guidance.^34^ An incremental cost-consequences analysis will document all intervention costs and consequences compared with the control. Several sensitivity analyses will be run to explore the sensitivity of results to changes in parameters including intervention costs.

#### Process evaluation

Recruitment, attendance, completion data and participant baseline characteristics will be summarised using descriptive statistics (means, SD and proportions). Descriptive statistics will be used to summarise participant characteristics, responses from the attendance sheets, acceptability questionnaires and facilitator delivery proformas. Data will be input in Excel and analysed using analytic software, for example, SPSS. Descriptive summaries will present session attendance, number of sessions delivered and fidelity to key programme components, allowing identification of variation and patterns of incomplete delivery. Differences in baseline characteristics between those who participated, those who did not and those who discontinued will also be examined to identify patterns of engagement.

Qualitative data from participant interviews (including non-completers), facilitator interviews and focus groups will be audio recorded, transcribed verbatim, deidentified and uploaded into NVivo software for analysis. Field notes from observations will be incorporated to provide additional insights into fidelity and participant engagement. A thematic framework approach^35^ will be used to explore participant experiences, mechanisms of change and contextual factors influencing delivery and sustained cycling.

Following separate quantitative and qualitative analyses, findings will be compared and integrated across data sources. This triangulation will allow examination of how context, implementation and mechanisms of action influenced trial outcomes and will help identify barriers and facilitators to delivery, engagement and long-term behaviour change.

### Monitoring

Given the low-risk nature of the trial and absence of early stopping criteria, a separate data and trial monitoring committee is deemed unnecessary.

### Ethics

#### Research ethics approval and amendments

Ethical approval has been obtained from the University of Glasgow Medical, Veterinary and Life Sciences Research Ethics Committee (number: 200240325, date 11 April 25). Any protocol amendments will be recorded on clinicaltrials.gov.

#### Confidentiality and data sharing

Personal data (contact details, e-consent forms) will be stored on a secure University of Glasgow server and deleted poststudy using secure software. Cycling organisations will be given participant names and phone numbers for session management and WhatsApp setup with a data sharing agreement in place.

#### Public and stakeholder involvement

The Cycle Nation intervention was co-developed with stakeholders, including HSBC UK staff, managers and representatives from HSBC and British Cycling, then adapted for communities in Manchester and Glasgow. Practical input on delivery has been provided by participating cycling organisations. A stakeholder advisory group meets annually, comprising representatives from Glasgow City Council, Sustrans, Bike for Good, Adaptive Riders Collective and Cycling Scotland.

### Limitations

This study will use self-reported outcome measures which may introduce reporting and recall bias but have been chosen because objective measures (eg, accelerometers) cannot reliably capture cycling behaviours. Bias will be minimised for secondary outcomes by using validated questionnaires. Although sessions are offered across multiple organisations, locations and times to maximise reach, some participants may still be unable to attend. Attrition is expected over the 12 months follow-up. Retention will be supported through voucher incentives for completing all follow-up questionnaires and home visits, when required.

## Supplementary material

10.1136/bmjopen-2025-112768 online supplemental file 1

10.1136/bmjopen-2025-112768 online supplemental file 2
