# Supplementary material for: Effectiveness and cost-effectiveness of a 9 week multi-component cycling programme versus an existing single cycling training session: protocol for the Cycle Nation Communities randomised controlled trial
Source: BMJ Open. 2026 Mar 3;16(3):e112768. doi: 10.1136/bmjopen-2025-112768 (PMC12958902; doi:10.1136/bmjopen-2025-112768)
Supplement: online supplemental file 1 [file bmjopen-16-3-s001.pdf]

University of Glasgow | College of Medical, Veterinary & Life Sciences

Participant ID:

Cycle Nation: a randomised controlled trial in communities in Glasgow
Researchers: Dr Emma Lawlor, Prof. Jason Gill, Prof. Cindy Gray, Fernanda Gabler Trisotti, Prof Emma McIntosh, Prof Alex McConnachie
PARTICIPANT CONSENT FORM

Please initial box

1	I confirm that I have read and understand the information sheet for the above study. I have had the opportunity to ask questions and have had these answered satisfactorily.	
2.	I understand that I have equal chance of being allocated to either the Cycle Nation programme or Cycling Scotland "Introduction to roads" course	
3	I understand that my participation is voluntary and that I am free to withdraw at any time, without giving any reason. I understand that the information I have already provided will be retained and used in the study.	
4	I agree to complete a questionnaire about my cycling and other changes from taking part in the Cycle Nation programme on three occasions, including information about myself.	
5	I am happy for researchers to attend, observe and audio-record programme sessions.	
6	I understand that researchers may take photographs of programme sessions however, I can request to not be in them.	
7	I acknowledge that I will be referred to by pseudonym, and that my name and other information likely to identify me will be anonymised.	
8	I understand that the material will be treated as confidential and will be kept in secure storage.	
9	I understand that other researchers (including students) will have access to my anonymised information.	
10	I understand that my information will be used in reports, academic papers, books, conferences and other dissemination events.	
11	I understand that I say anything that poses a threat to myself or other people, the research team may have to tell relevant third parties. .	
12	I acknowledge the provision of a Privacy Notice in relation to this research project.	

	YES	NO
I agree to take part in the above study		
If I am allocated to the Cycle Nation programme, I am happy to discuss my experience of the programme in interviews		
I am happy to be contacted if I do not complete the programme so the research team can find out why		
I would like to receive a summary report of the study findings		

____________________ __________ ____________________

Name of participant Date Signature

____________________ __________ ____________________

Researcher Date Signature
