# Supplementary material for: Effectiveness and cost-effectiveness of a 9 week multi-component cycling programme versus an existing single cycling training session: protocol for the Cycle Nation Communities randomised controlled trial
Source: BMJ Open. 2026 Mar 3;16(3):e112768. doi: 10.1136/bmjopen-2025-112768 (PMC12958902; doi:10.1136/bmjopen-2025-112768)
Supplement: online supplemental file 2 [file bmjopen-16-3-s002.pdf]

Participant ID:

University of Glasgow | College of Medical, Veterinary & Life Sciences

Cycle Nation: a randomised controlled trial in communities in Glasgow
Researchers: Dr Emma Lawlor, Fernanda Gabler Trisotti, Prof. Jason Gill, Prof. Cindy Gray, Prof Emma McIntosh, Prof. Alex McConnachie

CYCLE FACILITATOR CONSENT FORM

Please initial box

1	I confirm that I have read and understand the information sheet for the above study. I have had the opportunity to ask questions and have had these answered satisfactorily.	
2	I understand that my participation is voluntary and that I am free to withdraw at any time, without giving any reason.	
3	I am happy for researchers to attend, observe, audio-record and take photographs of programme sessions.	
4	I am happy to be contacted to discuss my experience of the programme in an interview/focus group, and understand that this will be audio-recorded and typed out for analysis	
5	I acknowledge that I will be referred to by pseudonym, and that my name and any other information likely to identify me will be anonymised.	
6	I understand that my information will be treated as confidential and will be kept in secure storage.	
7	I understand that other researchers (including students) will have access to my anonymised information.	
8	I understand that my information will be used in reports, academic papers, books, conferences and other dissemination events.	
9	I understand that any criminal acts which come to light as a result of my participation in this study may have to be reported appropriately to the relevant authorities by the research team.	
10	I acknowledge the provision of a Privacy Notice in relation to this research project.	

	YES	NO
I agree to take part in the above study.	☐	☐
I would like to receive a summary report of the study findings.	☐	☐

____

Name of participant Date Signature

____________________________ ____________ ____________

Researcher Date Signature
