# Supplementary material for: Effectiveness and cost-effectiveness of a 9 week multi-component cycling programme versus an existing single cycling training session: protocol for the Cycle Nation Communities randomised controlled trial
Source: BMJ Open. 2026 Mar 3;16(3):e112768. doi: 10.1136/bmjopen-2025-112768 (PMC12958902; doi:10.1136/bmjopen-2025-112768)
Supplement: Supplementary file 3 [file bmjopen-2025-112768.reviewer_comments.pdf]

PEER REVIEW HISTORY

BMJ Open publishes all reviews undertaken for accepted manuscripts. Reviewers are asked to complete a checklist review form and are provided with free text boxes to elaborate on their assessment. These free text comments are reproduced below.

ARTICLE DETAILS

Title (Provisional)

Effectiveness and Cost-Effectiveness of a Nine-Week Multi-Component Cycling Programme Versus an Existing Single Cycling Training Session: Protocol for the Cycle Nation Communities Randomised Controlled Trial

Authors

Lawlor, Emma; Gabler Trisotti, María Fernanda; McIntosh, Emma; McConnachie, Alex; Gill, Jason; Gray, Cindy

---

VERSION 1 - REVIEW

**Reviewer**	**1**
**Name**	**Pattanaprateep, Oraluck**
**Affiliation**	**Mahidol University Faculty of Medicine Ramathibodi Hospital, Department of Clinical Epidemiology and Biostatistics**
**Date**	**02-Nov-2025**
**COI**	**None**

---

clear and interesting protocol... thank you

**Reviewer**	**2**
**Name**	**Heron, Neil**
**Affiliation**	**Queen's University Belfast, General Practice**
**Date**	**23-Nov-2025**
**COI**	**None**

Dear Authors, Please consider the limitations of your study, how you will overcome these and include a section on this within the paper. Otherwise, well done on writing a nice protocol paper. Best wishes,

---

VERSION 1 - AUTHOR RESPONSE

Reviewer: 1
Dr. Oraluck Pattanaprateep, Mahidol University Faculty of Medicine Ramathibodi Hospital
Comments to the Author:
clear and interesting protocol... thank you

Thank you for your comment and reviewing the manuscript.

Reviewer: 2
Dr. Neil Heron, Queen's University Belfast, Keele University
Comments to the Author:
Dear Authors, Please consider the limitations of your study, how you will overcome these and include a section on this within the paper. Otherwise, well done on writing a nice protocol paper. Best wishes,

We have now added an additional section at the end of the manuscript entitled 'Limitations' – please see below for the added text within this section.

Limitations
This study will use self-reported outcome measures which may introduce reporting and recall bias but have been chosen because objective measures (e.g. accelerometers) cannot reliably capture cycling behaviours. Bias will be minimised for secondary outcomes by using validated questionnaires. Although sessions are offered across multiple organisations, locations, and times to maximise reach, some participants may still be unable to attend. Attrition is expected over the 12-months follow-up. Retention will be supported through voucher incentives for completing all follow-up questionnaires and home visits, when required.

Thank you for your comments and reviewing the manuscript.
